# Traditional medicinal plant use in Loja province, Southern Ecuador

**DOI:** 10.1186/1746-4269-2-44

**Published:** 2006-10-10

**Authors:** Rainer W Bussmann, Douglas Sharon

**Affiliations:** 1 University of Hawaii, Lyon Arboretum, 3860 Manoa Rd., Honolulu, HI 96822, USA; 2 Phoebe Hearst Museum of Anthropology, University of California Berkeley, USA

## Abstract

This paper examines the traditional use of medicinal plants in Loja province, Southern Ecuador.

Two hundred fifteen plant species were collected, identified and their vernacular names and traditional uses recorded. This number of species indicates that the healers, market vendors and members of the public interviewed still have a very high knowledge of plants in their surroundings, which can be seen as a reflection of the knowledge of the population in general. However, the area represents only an outlier of the larger Northern Peruvian cultural area, where more than 500 species of plants are used medicinally, indicating that in Ecuador much of the original plant knowledge has already been lost.

Most plant species registered are only used medicinally, and only a few species have any other use (construction, fodder, food). The highest number of species is used for the treatment of "magical" (psychosomatic) ailments (39 species), followed by respiratory disorders (34), problems of the urinary tract (28), Fever/Malaria (25), Rheumatism (23) and nervous system problems (20).

## Background

Traditional medicine or ethnomedicine is a set of empirical practices embedded in the knowledge of a social group often transmitted orally from generation to generation with the intent to solve health problems. It is an alternative to Western medicine and is strongly linked to religious beliefs and practices of indigenous cultures. Medicinal plant lore or herbal medicine is a major component of traditional medicine.

In Latin American countries, herbal medicine is deeply rooted, practiced extensively by indigenous groups, and frequently used by a broad cross-section of the larger society. Often it is an economically inevitable alternative to expensive Western medicine.

Knowledge is transmitted from one generation to the next by traditional healers, shamans or curanderos, and has survived the rigors of the Spanish conquest and extensive mestizaje or racial intermixing. Herbal medicine is, however, a dynamic phenomenon in constant evolution and additional knowledge has been acquired by natural selection over the centuries.

**Figure 1:**
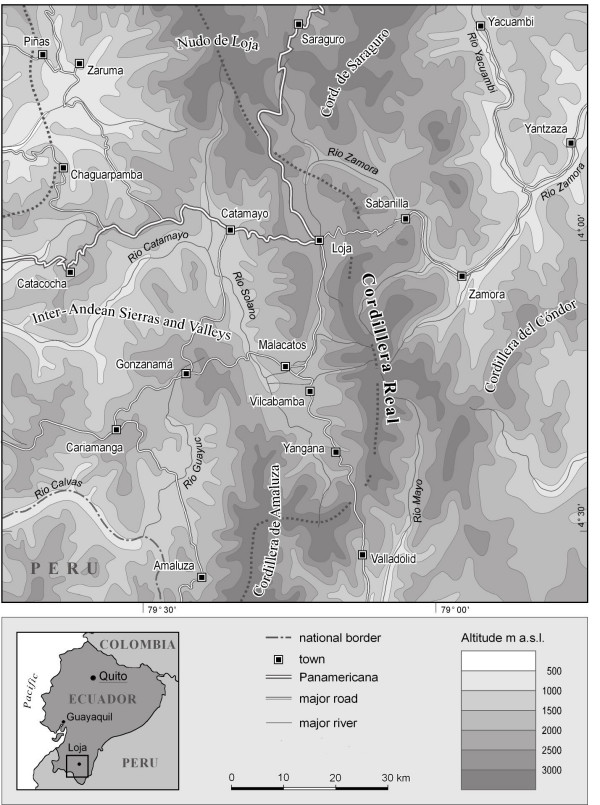
Study Area.

The border region of Ecuador and Peru (Fig. 1) is one of the most biologically diverse areas in the world, and thus is a "biodiversity-hotspot" par excellence. Low passes in the Andean chain allow an easy exchange between the flora and fauna of the Amazon Basin and the Pacific lowlands. Additionally, the region is characterized by a rapid transition between the humid mountain forests of the northern Andes and the dry, deciduous forests and desserts of the northern Peruvian lowlands.

Considerable progress has been made in the overall taxonomic treatment of the flora of the country as a whole [1-4]. However, the southern part of the country is relatively unexplored. The first floristic studies were conducted in the 1940's [5,6], followed by decades without any further research activity. Until the late 1990s little work had been done on vegetation structure, ecology, and ethnobotany.

A major lacuna in our knowledge of the Ecuadorian border region has to do with the rich shamanic lore found here, which has received little attention from anthropologists. Based upon 10 field trips to the southern side of the border in the late 1980s, Peruvian anthropologist Lupe Camino [7] has postulated a "health axis" of Andean ethnomedicine stretching from Loja, Ecuador in the highlands to the coastal desert of the Department of Piura in Northern Peru. Departing from the notion of "health coordinates," she defined this axis as a geographic space determined by shared concepts of health, specifically the "hot-cold" folk medical system found by anthropologists in many parts of Latin America. According to this theory, illness is explained as due to "cold" causes, such as entry of air into the body, or "hot" causes, such as excessive consumption of hot foods, with curing conforming to a doctrine of opposites: hot remedies to drive out cold and cold remedies to extract heat. The classic study of the "hot/cold dichotomy" was conducted by George Foster [8], who traced its origins to Greek humoral pathology brought by Spain to the New World. In contrast with this view, Camino sees the Andean folk medical system as rooted in the indigenous concept of "complementary opposites," and broadly applied to geography and cultural identity as well as the body, foods, and illness.

The present work grew out of an interdisciplinary project, initiated in 1995 on a grant from the San Diego Museum of Man, which involved collaboration between an ethnobotanist (Bussmann), a medical anthropologist (Sharon), an ethnophramacologist (Ezra Bejar) and a *curandero *(Cruz Roa) from the region around the town of San Pedro de Vilcabamba near Loja, Ecuador. The results of the preliminary fieldwork [9,10] were also published in a bilingual fieldguide [11], which included photos of the collected herbarium specimens, vernacular names, botanical identifications, descriptions as well as information on plant origin, ecological context, and indigenous uses and administration. Antecedents for this volume included [12] and [13] for the northern highlands of Ecuador, and [14] for the central highlands. In the current article this earlier work is incorporated into subsequent fieldwork and the entire corpus (215 plant species) is characterized in terms of indigenous nomenclature and medicinal usage.

Since the start of the project in 1995, there have been some relevant innovations. In 1996, the Royal Danish Academy of Sciences and Letters published a comprehensive work on the ethnobotanical use of plants by three indigenous peoples of Coastal Ecuador [15]. In May of 1997, Douglas Sharon – following a precedent established with the help of a Peruvian *curandero*, Eduardo Calderón at the end of the 70s – was invited to join a group of Ecuadorian scholars in teaching a course on traditional medicine at the Faculty of Medical Sciences of the University of Loja. Also as of August 10, 1998 article 44 of the new Constitution of Ecuador stated that the Republic "will recognize, respect, and promote the development of traditional and alternative medicine."

## Materials and methods

### Plant collections

The majority of plants were collected during field visits in August-September 1995, May 1996, August-November 1996, March 1997, and June-July 1997. The specimens were registered under the collection series "Bejar" and "CORD" (see additional file 1).

Vouchers of all specimens were deposited at the Herbario Estación Científica San Francisco (ECSF), Herbario Loja (LOJA), Herbario Nacional de Ecuador (QCNE) and Herbario de la Pontificia Universidad Católica de Ecuador (QCA). The identification of the plant material was conducted entirely in Ecuador. No plant material was exported in any form whatsoever.

### Nomenclature

The nomenclature of plant families, genera and species follows the Catalogue of Vascular Plants of Ecuador [2]. Species were identified using the available volumes of the Flora of Ecuador [4], and reference material in the herbaria QCA, LOJA and QCNE.

### Ethnobotany

Ethnobotanical data were collected while accompanying four local healers (*curanderos*) and three midwives (*parteras*) from the Loja, Vilcabamba, Catamayo, Palanda and Amaluza areas of Loja province, when they went into the field for harvesting and to the markets to buy plants. In addition, *curanderos *and *parteras *were visited in their homes during the ten months of fieldwork to observe the preparation of remedies, and the authors participated in multiple healing rituals with each healer. Plant uses were discussed in detail with informants, after seeking prior informed consent from each respondent. Following a semi-structured interview technique [16,17], respondents were asked to provide detailed information about the vernacular plant name in Spanish or Quichua; ailments for which a plant was used; best harvesting time and season; plant parts used, mode of preparation and application; and specific instructions for the preparation of remedies, including the addition of other plant species. In addition, market vendors in the areas named above, as well as other members of the public were asked about their plant use. All interviews were carried out in Spanish, with at least one of the authors present. Both authors are fluent in Spanish, and no interpreter was needed to conduct the interviews.

Data on plant species, family, vernacular name, parts used, traditional use and modality of use were recorded and are given in Additional File 1.

## Results

### Indigenous nomenclature

The vernacular names of the 215 registered plants used in Southern Ecuador are almost entirely derived from Spanish roots. Other indigenous languages, Quichua in particular, have no importance whatsoever in traditional plant nomenclature. Plants are compared to European introductions, and then named accordingly. Most species were named with only one vernacular name widely used by the healers involved in the study. This high uniformity of vernacular names indicates that most of the plants are well known and widely used in the region.

### Informant consensus

The species reported in this paper are widely known and are employed for a large number of medical conditions. The same plants are frequently used by a variety of healers for the same purposes, and with only slight variations in recipes. All species found were well known to the healers involved in the study, and were often easily recognized by their vernacular names by other members of the population. This indicates that these remedies have been in use for a long time by many people.

### Plant uses

A total of 215 taxa belonging to 158 genera and 76 families are now on record. Of these, 214 could be identified, most of them to the species level. A detailed overview of all plants encountered, their scientific and vernacular names, and all uses, is given in Additional File 1. This number of plants used represents only a fraction (about 5%) of the flora of the region. The families best represented are Asteraceae with 32 species, Euphorbiaceae, Lamiaceae and Solanaceae (11 species each), and Apiaceae, Fabaceae, Lycopodiaceae (9 species each) (Additional File 1). One hundred eighty-two (85%) of the species used are Dicotyledons, 20 Monocotyledons (9.3%), 12 ferns (5.5%), and one unidentified lichen was used.

One hundredseventy-nine species (83%) are indigenous to Southern Ecuador, while 36 species (17%) are introductions (Table 1). Many of the introduced species are medicinal plants brought in during colonial times.

**Table 1:** Main plant groups used in Southern Ecuador and plant origin

	**Number of species**	
		%
**Dicotyledoneae**	182	85
**Monocotyledoneae**	20	9.3
**Pteridophyta**	12	5.5
**Lichenes**	1	0.2
	**215**	**100**
**Indigenous**	**179**	**83**
**Introduced**	**36**	**17**

## Medicinal

Two hundred fifteen plants registered in Southern Ecuador had medicinal properties. The same species might be used for various medical conditions. In addition, the same medical condition (e.g. heart problems) might be treated using different plant parts and/or involved different applications, e.g., topical and oral. In the following the number of applications and the number of species used (in italics) are given to emphasize the importance of the treatment of specific conditions. The highest number of species (*39, 18.1%*) was used for the treatment of "magical" (psychosomatic) ailments, with fifty-nine applications (12.4% of all plant uses). Fever/Malaria (48 applications, *25 species*), respiratory disorders (45 applications, *34 species*), rheumatism (28 applications, *23 species*), and nervous system problems (24 applications, *20 species*) followed. Table 2 lists species used medicilinally, while Table 3 gives an overview of all illnesses treated.

**Table 2:** Plant families used for medicinal purposes in the study area in Southern Ecuador

	**Number of species**	
		%
**ASTERACEAE**	32	15
**EUPHORBIACEAE**	11	5.1
**LAMIACEAE**	11	5.1
**SOLANACEAE**	11	5.1
**APIACEAE**	9	4.2
**FABACEAE**	9	4.2
**LYCOPODIACEAE**	9	4.2
**AMARANTHACEAE**	8	3.75
**URTICACEAE**	7	3.25
**POACEAE**	6	2.8
**PIPERACEAE**	5	2.35
**VALERIANACEAE**	5	2.35
**GERANIACEAE**	4	1.85
**BROMELIACEAE**	3	1.4
**CACTACEAE**	3	1.4
**ERICACEAE**	3	1.4
**LAURACEAE**	3	1.4
**LYTHRACEAE**	3	1.4
**ORCHIDACEAE**	3	1.4
**ACANTHACEAE**	2	0.93
**APOCYNACEAE**	2	0.93
**BORAGINACEAE**	2	0.93
**BRASSICACEAE**	2	0.93
**GENTIANACEAE**	2	0.93
**MALVACEAE**	2	0.93
**MYRTACEAE**	2	0.93
**ONAGRACEAE**	2	0.93
**PAPAVERACEAE**	2	0.93
**PLANTAGINACEAE**	2	0.93
**PROTEACEAE**	2	0.93
**ROSACEAE**	2	0.93
**RUTACEAE**	2	0.93
**ACTINIDIACEAE**	1	0.46
**ADIANTACEAE**	1	0.46
**AGAVACEAE**	1	0.46
**ANACARDIACEAE**	1	0.46
**ANNONACEAE**	1	0.46
**AQUIFOLIACEAE**	1	0.46
**ARECACEAE**	1	0.46
**ASCLEPIADACEAE**	1	0.46
**ASPHODELACEAE**	1	0.46
**BASELLACEAE**	1	0.46
**BIGNONIACEAE**	1	0.46
**BOMBACACEAE**	1	0.46
**BURSERACEAE**	1	0.46
**CAMPANULACEAE**	1	0.46
**CANNACEAE**	1	0.46
**CAPRIFOLIAEAE**	1	0.46
**CARYOPHYLLACEAE**	1	0.46
**CELASTRACEAE**	1	0.46
**CHENOPODIACEAE**	1	0.46
**CYPERACEAE**	1	0.46
**EQUISETACEAE**	1	0.46
**IRIDACEAE**	1	0.46
**LICHENES**	1	0.46
**LILIACEAE**	1	0.46
**LOGANIACEAE**	1	0.46
**LORANTHACEAE**	1	0.46
**MIMOSACEAE**	1	0.46
**MYRISTICACEAE**	1	0.46
**NYCTAGINACEAE**	1	0.46
**OXALIDACEAE**	1	0.46
**PASSIFLORACEAE**	1	0.46
**POLEMONIACEAE**	1	0.46
**POLYGONACEAE**	1	0.46
**POLYPODIACEAE**	1	0.46
**PORTULACACEAE**	1	0.46
**PUNICACEAE**	1	0.46
**RUBIACEAE**	1	0.46
**SALICACEAE**	1	0.46
**SAPINDACEAE**	1	0.46
**SCROPHULARIACEAE**	1	0.46
**SELAGINELLACEAE**	1	0.46
**TILIACEAE**	1	0.46
**VERBENACEAE**	1	0.45
**ZINGIBERACEAE**	1	0.46

**TOTAL**	**215**	**99.97**

**Figure 2:**
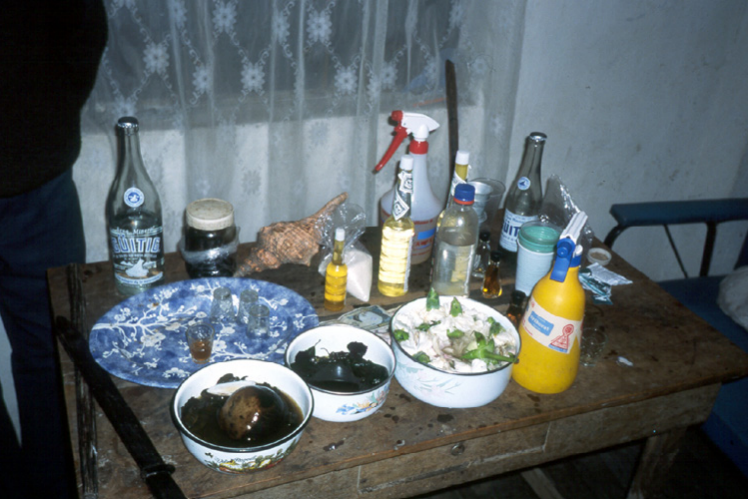
Ecuadorian "Westernized" healing *mesa*.

**Figure 3:**
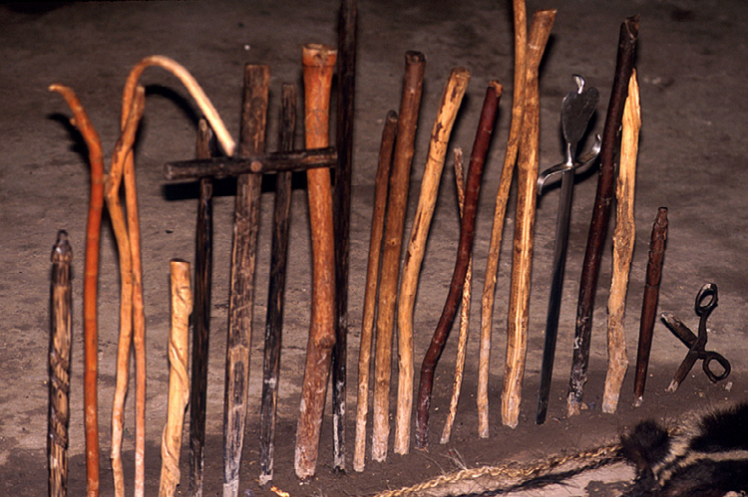
Part of traditional Peruvian *mesa*.

Most treatments are performed in the homes of the individual healers, who normally have their *mesas *(healing altars) already set up. In most cases in Southern Ecuador, a "Western" altar without many power objects is employed (Fig. 2), in contrast to Northern Peru, where normally a "traditional" *mesa *is set up (Fig. 3). This difference is rooted in the fact that traditional healing was illegal in Ecuador until the constitutional change of 1998. Additionally, traditional cures are often performed outdoors, either close to sacred lagoons or waterfalls, or at special ceremonial sites. A curing ceremony normally involves purifications of the patient by orally spraying blessed and enchanted herbal extracts on the whole body to fend off evil spirits.

Eighty-five different medicinal conditions were recorded. Most plants were used for the treatment of multiple ailments. The large variety of applications was grouped into 37 main categories (Table 3).

**Table 3:** Plant uses in the study area in Southern Ecuador

	**Number of uses**		**Number of species used**	
		%		%
**Magical**	59	12.42	39	18.1
**Fever, Malaria, Yellow fever**	48	10.10	25	12.6
**Respiratory**	45	9.47	34	15.8
**Urinary Tract**	40	8.42	28	13.0
**Rheumatic**	28	5.90	23	10.6
**Nerves**	24	5.05	20	9.3
**Liver**	23	4.84	19	8.8
**Stomach**	23	4.84	22	10.2
**Inflammation**	20	4.21	20	9.3
**Diarrhea and vomiting**	18	3.79	18	8.3
**Gynaecological**	16	3.34	16	7.4
**Heart**	16	3.34	16	7.4
**Food, Spice, Nutrition**	13	2.74	13	6
**Headache, Pain, Toothache**	13	2.74	13	6
**Wounds**	12	2.53	12	5.6
**Infections**	11	2.32	11	5.1
**Purgative**	8	1.68	8	3.7
**Skin problems**	6	1.26	6	2.8
**Construction**	6	1.26	6	2.8
**Fodder**	5	1.05	5	2.3
**Cramps**	5	1.05	5	2.3
**Bleeding**	4	0.85	4	1.86
**Eyes**	4	0.85	4	1.86
**Parasites**	4	0.85	4	1.86
**Blood purification**	3	0.64	3	1.4
**Diabetes**	3	0.64	3	1.4
**Tumors, Cancer**	3	0.64	3	1.4
**Bruises**	3	0.64	3	1.4
**Veterinary**	2	0.43	2	0.93
**Insect bites**	2	0.43	2	0.93
**Hernia**	1	0.22	1	0.46
**Gallbladder**	1	0.22	1	0.46
**Fractures**	1	0.22	1	0.46
**Ulcers**	1	0.22	1	0.46
**Allergies**	1	0.22	1	0.46
**Gangrene**	1	0.22	1	0.46
**Leucorrhea**	1	0.22	1	0.46
**Venereal disease**	1	0.22	1	0.46

**TOTAL**	**475**	**100**	**215**	

### Internal organs

The highest number of species was used to treat internal organ and digestive system disorders (101, 21% of all conditions treated). This included mostly urinary tract and kidney infections (40 applications, *28 species*, 13% of all species used), liver problems (23 applications, *19 species*) and stomach ailments, including ulcers (23 applications, *23 species*). Eighteen species (18 applications) were used for the treatment of diarrheic conditions. The use of medicinal plants for the treatment of gastrointestinal disorders has a high prevalence in other Andean societies was documented by a variety of studies [17-20].

### Magical

*Mal aire *(bad air), *mal viento *(bad wind), *susto *and *espanto *(fright), *mal ojo *(evil eye) and *envidia *(jealousy) are seen as very common illnesses in Andean society. Causes are sudden changes in body temperature, any kind of shock, spells cast by other people, poisoned food, etc. Medicinal problems caused by outside influence were reported by a wide variety of studies [e.g. [22-24]]. The western concept of "psychosomatic disorders" describes these illnesses.

Fifty-nine applications fell into the "magical" category, with *39 *plant species named to treat these disorders. *Mail aire *(18 applications), *susto *(14), and *sorcery *(9) were the most common magical illnesses encountered. Treatment in many cases involved the participation of the patient in a cleansing ceremony (*limpia*). This could either be a relatively simple spraying with perfumes and holy water, or a whole night ceremony involving the healers curing altar (*mesa*). In addition, patients frequently receive *seguros *(herbal amulets) for protection against further evil influences and for good luck. *Seguros *are flasks filled with powerful herbs as well as perfumes, pictures of saints, and hair and fingernails of the patient.

### Fever, Malaria, Yellow Fever

In many cases, any fever is attributed to malaria or yellow fever, although frequently no confirming diagnosis is conducted. Yellow fever is very rare in Southern Ecuador. The frequent occurrence of the term in local illness categories is thus somewhat surprising. Malaria is rather common in some parts of Loja province, and does indeed represent a serious threat to the population, especially during the rainy season. Therefore it is not surprising that *25 *plants were used to treat these conditions (48 applications).

### Respiratory

In many rural areas, the smoke of cooking stoves still escapes through the roof or doorway. Consequently, a large variety of respiratory problems is very common. Houses are also often very damp and cold, especially at higher altitudes. This leads to a high incidence of respiratory infections. Forty-five applications included respiratory ailments, with *34 *plant species employed to treat respiratory conditions. The most prevalent respiratory problems were the common cold (21 applications), cough (8), flu (7), and bronchitis (2).

### Rheumatic problems

Twenty-eight applications comprised rheumatic problems, with *23 *plant species used to treat rheumatic and musculo-skeletal ailments. Most of these arise from the living conditions of the population, mainly damp and cold caused by insufficient insulation, heating, and circulation in rural houses. Rheumatic conditions include arthritis, rheumatic fevers, muscular and skeletal pains, as well as body-joint pain.

### Nerves

A fair number of species (*20*, for 24 applications) was used to treat nervous system disorders. This includes general nervous disorders (11 applications), depression (5) as well as psychological fatigue (4).

### Inflammation

General inflammation of the body, without distinguishing if caused by natural or "magical" causes was treated with *20 *plant species.

### Gynecological problems

Menstruation problems, and complications in childbirth are very common medical conditions in Southern Ecuador. Sixteen plants were employed to treat these disorders, with six species used to cure vaginal infections, and four species each for the treatment of childbirth complications and menstrual regulation.

### Heart and circulatory system

The main application for circulatory system problems was the treatment of heart pain. Twelve species were used for the treatment of heart conditions, including heart attacks and heart pain. Three species were used to regulate hypertension, and one species helped to lower cholesterol levels.

### Pain

Thirteen species were used as analgesics, especially for the treatment of headaches, general pain, and toothache.

### Infection (bacterial and viral)

Bacterial and viral infections, especially wounds, were a major concern. Twelve plant species were used, mostly as poultices, to treat infected wounds, one of them to treat gangrene. Eleven species were used to treat internal bacterial infections.

### Other uses

A wide variety of plants were used to treat other ailments: Eight species served as purgatives, five were used to remedy cramps. Hemorrhages, eye infections, skin disorders, and parasites (amoebas and worms) were treated with four species each.

Three species were used for blood purification, diabetes, and cancer. Insect bites (2), hernia, fractures, allergies, leucorrhea and venereal disease (one species each) were less important medical conditions treated.

## Parts of medicinal plants used and mode of application

In most cases (61%) the whole plant was used for medicinal purposes, followed by leaves (13%), and flowers (6%), the seeds, roots, bark, fruits, and latex were rarely used (3% each) (Table 4).

**Table 4:** Plant part used for medicinal purposes

**Plant Part**	**Number of uses**	
		%
**Whole plant**	146	61.1
**Leaves**	32	13.5
**Flowers**	15	6.3
**Seeds**	8	3.3
**Root**	8	3.3
**Bark**	8	3.3
**Fruit**	8	3.3
**Latex**	7	2.9
**Wood**	5	2.1
**Branches**	2	0.9

** TOTAL**	**239**	**100**

Almost all remedies were prepared from fresh plant material (96%) (Table 5). All of the introduced plant species were cultivated in fields and gardens, while most of the indigenous species were collected in the wild.

**Table 5:** Plant constitution

**Constitution**	**Number of uses**	
		%
**Fresh**	207	95.8
**Dry**	9	4.2

** TOTAL**	**216**	**100**

Diseases and other health problems were most frequently treated with decoctions of various plant species. The use of single species for treatments was rare.

Of all preparations mentioned, plants were mostly boiled in water or sugarcane alcohol (*aguardiente*).

The most frequent way to administer remedies was as to prepare a decoction and ingest it orally (67.8%), followed by application as a poultice (31.8%, plant crushed or boiled and applied). Only 0.4% of the plants were burned for inhalation (Table 6).

**Table 6:** Preparation and application methods for medicinal plants:

**Application**	**Number of uses**	
		%
**Oral**	168	67.8
**Topical**	79	31.8
**Inhalation**	1	0.4

** TOTAL**	**248**	**100**

## Food and spices

Thirteen plant species, predominantly European introductions like *Pimpinella anisum, Foeniculum vulgare, Origanum vulgare*, etc. were used as food and spices, in addition to their medicinal use.

## Construction

*Erythrina *and *Inga *species were used as material to plant live fences. *Ochroma pyramidale *(*balsa*) was used as a very light construction timber. Palm fronds and palm stems (*Bactris *sp.) were used for thatching and roof construction.

## Ceremonial

Palm staffs (*Bactris *sp.) are still used as power objects on Southern Ecuadorian *mesas*.

## Veterinary and fodder

Hardly any plants in Southern Ecuador had veterinary uses. *Cicuta virosa *was used to treat animal wounds. Various species of *Erythrina *and *Inga *were used as animal fodder. The introduced *Melinis minutiflora *was mostly used as fodder.

## Conclusion

Andean societies have used plants for physical therapy and psychosomatic ailments for millennia. Southern Ecuador falls into the Northern Peruvian cultural area. It appears to represent a Diaspora from the latter area, and a region where traditional plant knowledge, though important, has declined considerably.

The use of hallucinogens, in particular the San Pedro cactus (*Echinopsis pachanoi*) is a vital component in Andean healing practices, and has been practiced for millennia [25-28]. However, Southern Ecuadorian *curanderos *and *parteras *have almost entirely abandoned this custom. In fact, San Pedro was not mentioned as a mind-altering plant by any healer or midwife interviewed, and was not used in curing ceremonies. Centuries of prohibition have led to a pronounced abandonment of traditional knowledge. This is also reflected in the current study. Many plants used for "magical" purposes in Peru [29] have disappeared from traditional use in Ecuador. The fear of prosecution is still very deeply rooted in the healer community, and most healers interviewed stated that they did not wish to be cited by name.

Most healing altars or *mesas *in Southern Ecuador are almost entirely devoid of any "pagan" objects such as seashells, pre-Columbian ceramics etc. Patients are cleaned, by spraying them with holy water and perfumes. In rare cases tobacco juice and extracts of Jimson weed (*Datura ferox*) are used to purify the patients. Southern Ecuadorian *mesas *are also much less elaborate than the *mesas *of Peruvian *curanderos *(Figs. 2 and 3). The incantations used by healers during their curing sessions center on Christian symbolism. References to Andean cosmology are almost entirely absent, and the use of guinea pigs as diagnostic instruments has all but disappeared from the tool kit of these healers.

Interestingly, Peruvian *curanderos *have started to fill this spiritual void in Southern Ecuador. Healers from the Northern Peruvian mountains and coastal plains frequently cross over to Ecuador to offer their services to patients – including increasing numbers of foreigners with a "New Age" orientation – who are not satisfied with the more Westernized approach of Ecuadorian healers. These Peruvian colleagues have a much more elaborate plant knowledge, and their *mesas *as well as their incantations follow a more traditional pattern.

For the most part, the knowledge of medicinal plants is still transmitted orally. An illustrated identification guide for Southern Ecuadorian plant use [11] will hopefully help to keep the traditional knowledge left in this area alive.

## Authors' contributions

Both authors share the contributions to fieldwork, data analysis, and compilation of this manuscript.

## Declaration of competing interests

The author(s) declare that they have no competing interests.

## Supplementary Material

Additional file 1 Medicinal plant species of Southern Ecuador: Scientific and vernacular names, uses and preparation. The data provided represent the complete overview on all plants encountered: Scientific names, vernacular names, plant parts used, preparation and uses. Click here for file
