# Supplementary material for: Traditional medicinal plant use in Loja province, Southern Ecuador
Source: J Ethnobiol Ethnomed. 2006 Oct 10;2:44. doi: 10.1186/1746-4269-2-44 (PMC1615866; doi:10.1186/1746-4269-2-44)
Supplement: Additional file 1 — Medicinal plant species of Southern Ecuador: Scientific and vernacular names, uses and preparation. The data provided represent the complete overview on all plants encountered: Scientific names, vernacular names, plant parts used, preparation and uses. [file 1746-4269-2-44-S1.pdf]

Additional file 1. Species encountered and used in Southern Ecuador						
Family/Species	Indigenous name	Plant part used	Admin.	Preparation	Use	Coll. #
ACANTHACEAE						
Justicia sp.	Hierba de la Justicia	Whole plant, fresh	Oral	Cooked with sugar.	Heart, Pneumonia, Anxiety, Psychological tension, Fright / Susto, Freight in children / Espanto	Bejar148
Sanchezia oxysepala Mildbr.	Cimora Toro	Flowers and small Leaves, fresh	Oral	Put Flowers into flask with alcohol.	Against sorcery and for dreams, for good luck	Bejar76
ACTINIDIACEAE						
Saurauia spp.	Hierba del Oso	Leaves, fresh	Topical	Applied to the affected area.	Rheumatism	Bejar152
ADIANTACEAE						
Adiantum concinnum Wild. ex H.B.K.	Cucharillo	Whole plant, fresh	Oral	Cooked with sugar.	Diabetes	Bejar92
AGAVACEAE						
Agave americana L.	Cabuyo, Penca de Caballo	Leaves, fresh	Topical	Parboiled.	Depression, Curse someone, Defend against sorcerers during sessions	Bejar48, 236
AMARANTHACEAE						
Aerva lanata (L.) Jus.	Escancel	Flowers, fresh	Topical	Fresh or cooked with Flor de Malva tea and Aloe juice, rubbed.	Infection, Depression, Liver, Headache	Bejar128
Alternanthera porrigens (Jacquin) Kuntze	Moradilla	Whole plant, fresh	Oral	Cooked with sugar, Malva Olorosa, Toronjil.	Kidneys, Lungs	Bejar200
Amaranthus cruentus L.	Sangorache	Whole plant, fresh	Oral	Horchata with Aguardiente (Sugarcane spirit).	Headache, Fever, Gangrene, Specific female illnesses	CORD18
Amaranthus hybridus L.	Bledo	Whole plant, fresh	Oral	Horchata, 1/2 Tsp in 1L Horchata.	General pain, Heat in the breast	CORD8
Amaranthus retroflexus L.	Bledo Serrano, Cancel Serrano	Whole plant, fresh	Oral	Horchata, 1/2 Tbsp in 1L of water for horchata.	Flu, Headache, Fever	CORD37
Amaranthus spinosus L.	Bledo	Whole plant, fresh	Oral	Boiled and eaten as soup.	Food	Bejar44
Amaranthus sp.	Lancetilla	Whole plant, fresh	Oral	Horchata, take 1/2 Tbsp for 1 liter of water.	Flu, Insect bites	CORD14
Iresine diffusa H.B.K. ex Willd.	Sangrinaria	Whole plant, fresh	Oral	Horchata, 1 Tbsp in 1L Horchata.	Vaginal discharge with blood	CORD43
ANACARDIACEAE						
Schinus molle L.	Molle	Leaves and Seeds, fresh	Topical, Oral		Rheumatism, Spice	Bejar196
ANNONACEAE						
Annona cherimola Miller	Cherimoya	Seeds	Oral	Boiled.	Cold, during childbirth	Bejar110

Family/Species	Indigenous name	Plant part used	Admin.	Preparation	Use	Coll. #
APIACEAE						
Apium graveolens L.	Apio Negro	Whole plant, fresh	Oral	Boiled, taken with sugar for 15 days.	To reduce swellings	Bejar36
Cicuta virosa L.	Cicuta	Whole plant,. fresh	Topical	Boiled with Chila Chilo, Cordoncillo, as Poultice, washing of wounds.	Wounds of animals	Bejar74
Coriandrum sativum L.	Culantro, Cilantro	Whole plant, fresh or dried	Oral	1 Tbsp in 1L Horchata.	Sleeping aid, Spice	CORD62
Cyclospermum leptophyllum (Pers.) Eichler	Cuminillo	Whole plant, fresh	Oral	Cooked with Malva Olorosa and sugar.	Diarrhea, Stomach	Bejar82
Daucus montanus H. & B. ex Spreng.	Culantrillo	Whole plant, fresh	Oral	1 Tbsp in 1L Horchata.	Stomachache, Gas	CORD45
Eryngium foetidum L.	Culantrillo	Whole plant, fresh	Oral	Boiled with Cola de Caballo, Matico (Leaves), Cadillo, Caña Agria, and Trensilla.	Kidneys	Bejar92
Foeniculum vulgare P. Miller	Hinojo	Whole plant, fresh	Oral	Boiled with sugar and Aguardiente.	Infections, Women after childbirth	Bejar164
Petroselinum crispum (Miller) A.W. Hill	Perejil	Leaves, fresh	Oral and Topical	Parboiled with meat to eat. with salt to make a poultice.	Infections, Nose bleeds, To forget love or trauma, Spice	Bejar240
Pimpinella anisum L.	Anis	Whole plant, fresh or dried	Oral	Cooked as tea.	Colds, Chills	Bejar34
APOCYNACEAE						
Plumeria rubra L.	Suche	Latex	Topical	Applied to the affected area with unwashed lamb's wool. patient must not eat oils or lard. Administer for 15 days.	Hernia	Bejar278
Thevetia peruviana (Pers.) Schum.	Camalonga	Bark and seeds, fresh	Oral	Decoction of the Bark or seed.	Fever, Heart, Against Evil Eye/Mal Ojo	Bejar54
AQUIFOLIACEAE						
Ilex guayusa Loes	Guayusa	Whole plant, fresh	Oral	Cooked, drunk with Lemon and Orange.	Sorcery, Diuretic, Anemia	Bejar144
ARECACEAE						
Bactris spp.	Chonta	Wood			Ceremonial staffs, Construction of houses and roofs	Bejar114
ASCLEPIADACEAE						
Marsdenia cundurango Rchb.f.	Condorango	Stems and Bark, fresh or dried	Oral	Soaked in Cane alcohol.	Kidneys	Bejar84
ASPHODELACEAE						
Aloe vera (L.) Burm f.	Sábila, Savila Penca	Whole plant, fresh	Oral	Take the crystals washed from 1 Leaves and mix with orange or honey in 1/2l of water. the acid is put on red inflammations.	Kidneys, Liver, Inflammation, Weight loss, Internal infections, Protecting the house	CORD33, Bejar274

Family/Species	Indigenous name	Plant part used	Admin.	Preparation	Use	Coll. #
ASTERACEAE						
Achyrocline halii Hieron	Vida Vida	Whole plant, fresh	Oral		Cough, Heart, Nerves, Epilepsy	CORD1
Ageratum conyzoides L.	Pedromera Blanca, Pedrorera Blanca	Whole plant, fresh	Oral	Cooked, mixed with Manzanilla or *Fleishmannia* sp. (Asteraceae).	Gastrointestinal problems	Bejar230, 232
Ambrosia peruviana Willd.	Altamís	Whole plant, fresh	Topical	1 fresh Leaves on the forehead.	Headache	CORD61
Artemisia absinthium L.	Ajenjo, Ajenco, Polén	Leaves, fresh	Oral, Topical	Cooked. Baths, with 3 Tbsp in 2L of hot water.	Body pain, Stomach Pain. only for critical illnesses	Bejar 25, CORD57
Baccharis obtusifolia H.B.K.	Chilca Redonda	Whole plant, fresh	Oral, Topical	Cooked, taken with sugar. For hot seated baths - 3 Tbsp in 2L of hot water.	Stomach Pain, Vomiting, Bad Air / Mal Aire, Colds, Bone pain	Bejar106, CORD28
Bidens pilosa L.	Huichinge	Whole plant, fresh	Oral	Cooked.	Liver, Infections, Diarrhea	Bejar168
Brickellia diffusa (Vahl.) A. Gray	Monte de la Huanchaca	Whole plant, fresh	Topical	Boiled with Sauco, Chin Chin, Chila Chilo, Hierba del Coche, Santa Maria, Suelda con Suelda and Aguardiente. rubbed externally.	Fright / Susto	Bejar198
Chrysanthemum leucanthemum L.	Margarita	Whole plant, fresh	Oral, Topical	Rubbed externally and placed in the mouth of the patient during cleansing ceremonies.	Bad Air / Mal Aire	Bejar190
Chuquiraga jusieui J.F. Gmel	Chuquiragua	Branches and Leaves, fresh	Oral	1/2 Tbsp in 1L of boiling water, taken with sugar.	Cold, Cough, Pain of the Bones, Flu, Malaria, Fever	Bejar120, CORD34
Chuquiraga spinosa sp. huamanpinta C. Ezcurra	Chuquiragua	Branches and Leaves, fresh	Oral	1/2 Tbsp in 1L of boiling water, taken with sugar.	Cold, Cough, Pain of the Bones, Flu, Malaria, Fever	Bejar120, CORD34
Conyza sp.	Saucillo	Whole plant, fresh	Oral	Horchata, 1 Tbsp of Flowers in 1L of Horchata.	Colic	CORD24
Dorobea pimpinellifolia (H.B.K.) B. Nord	Borraja Serrana	Whole plant, fresh	Oral	1 Tbsp in 1L Horchata.	Cough, Flu	CORD38
Eupatorium dendroides Spreng.	Chila Chilo	Whole plant, fresh	Topical	Bath prepared with Chin Cil, Hierba del Cocke, Monte de la Huanchaca.	Bad Air / Mal Aire, Fright (Children)/ Espanto	Bejar104
Gnaphalium americanum Mill.	Vira Vira, Lechuguilla	Whole plant,. fresh	Oral	Cooked, 1 Tbsp in 1L boiling water.	Bad Air / Mal Aire, Impact, shock, Diarrhea in children	Bejar302, CORD46
Loricaria thyrsoidea (Cuatr.) Dillon	Valeriana Fina, Trensilla	Whole plant, fresh	Oral	2 Tbsp in 1L boiling water or Aguardiente.	Cramps, Bone pain, Bad Air / Mal Aire, Kidneys	CORD50, Bejar290
Matricaria frigidum (HBK) Kunth	Manzanilla	Whole plant, fresh	Oral and Topical	1/2 Tbsp in 1L of water for horchata, with sugar, Aspirin, Caña Agria, Cola de Caballo, Cadillo and Culantrillo. with seeds and cocoa-butter for stomach ailments.	Stomach pain, Colds, Fever, Diarrhea, To wash skin	CORD23, Bejar186
Onoseris odorata (D. Don) Hooker & Arnott	Lechuguilla	Whole plant, fresh	Oral	Cooked with sugar, Hierba del Perro.	Diarrhea (Children)	Bejar176

Family/Species	Indigenous name	Plant part used	Admin.	Preparation	Use	Coll. #
Pentacalia sp.	Valeriana Fina	Whole plant, fresh	Oral	2 Tbsp in 1L boiling water or Aguardiente.	Cramps, Bone pain, Bad Air / Mal Aire, Kidneys	CORD51
Picrosia longifolia D. Don	Cerraja	Whole plant, fresh	Oral	Crushed and parboiled.	Liver, Amoebas	Bejar72
Porophyllum ruderale (Jacq.) Cas.	Hierba Gallinazo	Whole plant, fresh	Oral and Topical	With Aguardiente or as a poultice.	Bad Air / Mal Aire, Fright / Susto	Bejar156
Pseudelephantopsus spicatus (B.Jus. ex Aubl.) Rohr	Suelda con Suelda	Whole plant, fresh	Topical	Cooked with Hierba del Coche y Monte de la Huanchaca. Poultice.	Freight/ Susto (Children), Stomach Pain, Wounds	Bejar280
Pseudelephantopus spiralis (Lesing) Cronquist	Chicoria	Whole plant, fresh	Oral	Cooked.	Fever, High blood presure, Shivering fits, Malaria	Bejar102
Senecio sp.	Retama Serrana	Whole plant, fresh	Oral	Horchata, 1 Tbsp of Flowers in 1L of Horchata.	Yellow Fever	CORD39
Siegesbeckia orientalis L.	Pacunga	Whole plant, fresh	Oral	Cooked with Pedromera, Manzanill, Cominillo.	Stomach	Bejar220
Tanacetum parthenium (L.) Sch. Bip.	Santa Maria	Leaves, fresh	Topical	Rub externally with Aguardiente, Monte Gallinazo.	Fright / Susto, Witchcraft, Against the devil	Bejar266
Tagetes minuta L.	Chin Chil	Whole plant, fresh	Topical	Rubbing with Monte de Coche, Alcanfora, Chila Chilo, Aguardiente.	Skin irritation, Bad Air / Mal Aire, Fright (Children)/Espanto caused by the rainbow	Bejar108
Tagetes sp.	Chinininge	Whole plant, fresh	Oral	Mince and extract juice, 1 Tbsp for every chicken.	Inflamed gall bladder in chicken	CORD65
Taraxacum officinale Wiggers	Diente de Leon	Whole plant, fresh	Oral	Cooked, with Aguardiente.	Liver, High blood presure	Bejar124
Vernonia patens H.B.K.	Laritaco	Leaves, fresh	Oral	1 glass a day during the evening, 1 month.	Rheumatism, Bad Wind/ Mal Viento, Boils, Freight / Espanto	Bejar174
Werneria sp.	Clavel Amarillo de Sepa, Clavel Amarillo de Yuca	Whole plant, fresh	Oral	Horchata, 1/2 Tbsp in 1L of water for horchata.	Yellow Fever	CORD41
BASELLACEAE						
Basella alba L.	Lutuyuyo	Root, fresh	Oral	Boiled with sugar, Buenas Tardes and San Agustin Lluyo.	Diarrhea	Bejar180
BIGNONIACEAE						
Tecoma stans (L.) Jus. ex H.B.K.	Fresmo, Gualpe	Flowers, fresh or dried	Oral	2 Tbsp of Flowers in 1L Horchata.	Yellow Fever	Bejar134, CORD71
BOMBACACEAE						
Ochroma pyramidale (Cav. ex Lam.) Urban	Balsa	Wood			As very light construction timber	Bejar40

Family/Species	Indigenous name	Plant part used	Admin.	Preparation	Use	Coll. #
BORAGINACEAE						
Borrago officinalis L.	Borraja de jardín	Whole plant, fresh	Oral	Horchata, 2 Tbsp in 1L of boiling water.	Asthma, Cough, Bronchitis	CORD59
Heliotropium spp.	Yanacaspe	Whole plant, fresh	Topical	Crush, apply directly on tooth.	Toothache, Broken teeth	Bejar304
BRASSICACEAE						
Lepidium virginicum L.	La Chicera	Whole plant, fresh	Oral		Fright (Children)/ Espanto (niños), Protection during childbirth, Post-partum protection	Bejar172
Rorippa nasturtium-aquaticum (L.) Hayek	Berros	Whole plant, fresh	Oral	Crushed and cooked with milk.	Lungs	Bejar42
BROMELIACEAE						
Puya eryngioides André	Achupalla Amarilla	Whole plant, fresh	Oral	1 Tbsp in 1L boiling water.	Yellow Fever	CORD53
Puya maculata L.B. Sm.	Achupalla Negra	Whole plant, fresh	Oral	1 Tbsp in 1L boiling water.	Yellow Fever	CORD52
Puya sp.	Chupaya	Stems, fresh	Oral, Topical	Cooked, drunk with sugar or applied as ointment.	Tumors, Infections	Bejar118
BURSERACEAE						
Bursera graveolens (H.B.K.) Triana & Planchon	Palo de Santo	Whole plant, fresh	Oral, Topical	Extract juice, take with sugar. Latex rubbed or applied as a wash.	Anemia, Fragrance	Bejar226
CACTACEAE						
Cephalocereus royeni (L.) Britten & Rose	Pitayo	Fruits and Leaves, fresh	Oral	Extract juice and drink.	Liver, Witchcraft	Bejar248
Echinopsis pachanoi (Britton & Rose) Friedrich & G. Rowley	San Pedro, Huachuma	Whole plant, fresh	Oral	One glass daily to prevent ulcers.	Purgative, Ulcers	Bejar166
Opuntia pubescens Wendland	Gorondilla	Leaves, fresh and without spines	Oral	Boiled a drink together with Eucalipto, Cola de Caballo, Culantrillo, Matico, Prenadilla, Moraja.	Removes obstructions in the urinary tract	Bejar136
CAMPANULACEAE						
Centropogon sp.	Cholo Valiente	Whole plant, fresh	Topical	Parboiled with Aguardiente as poultice and bath.	Rheumatism, Pain of the Bones, Cold Feet, Wounds	Bejar112
CANNACEAE						
Canna indica L.	Achira Negra	Leaves, fresh	Topical	Pinch one Leaves and put the warm oil that drips out on the belly.	Insect bites	CORD29
CAPRIFOLIAEAE						
Sambucus peruviana HBK	Sauco Tilo	Whole plant, fresh	Oral	Horchata, 1/2 Tbsp in 1L of water for horchata.	Flu, Heart pain	CORD22

Family/Species	Indigenous name	Plant part used	Admin.	Preparation	Use	Coll. #
CARYOPHYLLACEAE						
Dianthus caryophyllus L.	Clavel	Flowers, fresh	Oral	Cooked with Toronjil.	Heart, Nervous system, Nasal passages	Bejar78
CELASTRACEAE						
Maytenus laevis Reiscek	Chuchuasi	Bark, fresh	Topical and Oral	Bark soaked for 15 days in Aguardiente, then ground and heated, applied as ointment and drunk.	Kidneys	Bejar116
CHENOPODIACEAE						
Chenopodium ambrosioides L.	Paico	Whole plant, fresh	Oral, Topical	Extract the juice of the Leaves, put 1 Tbsp in 1/2L of fresh water. For intelligence blend with an egg. With Hierba del Perro, very gentle for children. Rubbed externally.	Parasites, Intelligence	Bejar222, CORD32
CYPERACEAE						
Cyperus sp.	Dictamo del Campo	Root, fresh	Oral	Boiled and taken with sugar for 15 days.	Allergies	Bejar122
EQUISETACEAE						
Equisetum bogotense (H.B.K.) Kunth	Cola de Caballo	Whole plant, fresh	Oral	1 Tbsp in 1L Horchata, drink with Cadillo, Trensilla, Caña Agria, Culantrillo.	Kidneys, Washing of wounds	Bejar80, CORD9
ERICACEAE						
Bejaria aestuans L.	Payama Rosada	Whole plant, fresh	Oral	Horchata - 1 Tbsp in 1L water.	Kidneys	CORD2
Gaultheria reticulata H.B.K.	Pajama, Uva	Whole plant, fresh, Fruits	Oral	2 Tbsp in 1L of boiling water, Fruits and juice eaten.	Kidneys, Inflammation, Food	CORD47
Macleania salapa Bentham	Salapa	Whole plant, fresh, Fruits	Oral	Oralen as vegetable, Fruits, juice.	Nutritional supplement	CORD88
EUPHORBIACEAE						
Croton draconoides Muell. Arg.	Sangre del Drago	Latex, fresh	Oral	Extract and take.	Kidneys, Infections	Bejar264
Croton lechleri Muell. Arg.	Sangre del Drago	Latex, fresh	Oral	Extract and take.	Kidneys, Infections	Bejar264
Croton wagneri Muell. Arg.	Mosqueera	Leaves, fresh	Oral	Cooked with Pico Pico.	Stomach pain, Pneumonia, Rheumatism, Tooth extraction	Bejar204
Jatropa curcas L.	Piñon	1. Latex, fresh<br>2. Seeds	1. Topical.<br>2. Oral	1. Rubbed externally<br>2. 2-3 seeds.	1. Wounds<br>2. Purgative, causes vomiting and diarrhea	Bejar246
Jatropa gosypiifolia L.	Piñon	1. Latex, fresh<br>2. Seeds	1. Topical.<br>2. Oral	1. Rubbed externally<br>2. 2-3 seeds.	1. Wounds<br>2. Purgative, causes vomiting and diarrhea	Bejar246
Jatropa multifida L.	Piñon	1. Latex, fresh<br>2. Seeds	1. Topical.<br>2. Oral	1. Rubbed externally.<br>2. 2-3 seeds	1. Wounds.<br>2. Purgative, causes vomiting and diarrhea	Bejar246

Family/Species	Indigenous name	Plant part used	Admin.	Preparation	Use	Coll. #
Phyllanthus niruri L.	Chanca Piedra	Whole plant, fresh	Oral	2 Tbsp of Flowers in 1L Horchata, drink with honey.	Kidney stones, Stomach ache	CORD70, Bejar100
Phyllanthus stipulatus (Raf.) Webster	Chanca Piedra	Whole plant, fresh	Oral	2 Tbsp of Flowers in 1L Horchata, drink with honey.	Kidney stones, Stomach ache	CORD70, Bejar100
Phyllanthus urinaria L.	Chanca Piedra	Whole plant, fresh	Oral	2 Tbsp of Flowers in 1L Horchata, drink with honey.	Kidney stones, Stomach ache	CORD70, Bejar100
Ricinus communis L.	Higuerilla	Bark, fresh	Topical	Cooked with Aguardiente, for a poultice.	Fractures, Sprains	Bejar162
FABACEAE						
Calliandra taxifolia (Kunth) Benth	Seda Seda	Flowers, fresh	Oral	Tea, 2 Tbsp of Flowers in 1L boiling water, drink with honey.	Red vaginal discharge	CORD669
Desmodium molliculum (H.B.K.) DC.	Vijado Blanco	Whole plant, fresh	Oral	Horchata: take 1 Tbsp of the whole plant and put in 1L of water, add honey.	Intestinal pain, White and yellow vaginal discharge	CORD15
Desmodium triflorum (L.) DC	Pega Pega	Whole plant, fresh	Topical	Cooked, with Aguardiente, as poultice.	Lesions	Bejar234
Erythrina fusca Loureiro	Porotillo	Bark, wood and Leaves	Oral	Feed.	Fodder, Fencing	CORD86
Erythrina velutina Willdenow	Porotillo	Bark, wood and Leaves	Oral	Feed.	Fodder, Fencing	CORD86
Inga edulis C. Martius	Pajúl, Guato	Whole plant	Oral	Feed.	Fodder for pigs, Fencing	CORD87
Inga feuillei DC.	Pajúl, Guato	Whole plant	Oral	Feed	Fodder for pigs, Fencing	CORD87
Lupinus sp.	Vijado Serrano	Whole plant, fresh	Oral	Horchata, 1/2 Tbsp in 1L of water for horchata.	Inflammation, White vaginaldischarge	CORD42
Mimosa acantholoba (Humb. & Bonpl. ex Willd.) Poir	Uña de Gato	Whole plant, fresh	Oral and Topical	Crushed and boiled, applied as poultice or drunk.	Cancer	Bejar296
Spartium junceum L.	Retama	Whole plant, fresh	Oral	2 Tbsp in 1L of Aguardiente, to clean.	Freight/ Susto	CORD30
GENTIANACEAE						
Centaurium erythraea Rafin	Canchalagua	Whole plant, fresh	Oral	Cooked with also used sugar, Huichinge and Verbena. Comparable to Chuquiragua.	Cold, Cough, Flu	Bejar62
Halenia weddeliana Gilg.	Clavel Amarillo Seco	Whole plant, fresh	Oral	1 Tbsp in 1L Horchata.	Yellow Fever	CORD36
GERANIACEAE						
Geranium humboldtii Sprengel	Valeriana	Whole plant, fresh	Topical	3 Tbsp in 1L Aguardiente, then spit at children.	Inflammation, Cramps, Evil Air / Mal Aire	CORD44
Pelargonium odoratisimum (L) L' Herit ex Ait	Malva Olorosa	Whole plant, fresh	1. Oral<br>2. Topical	1. Horchata: 1 Tbsp in 1L water<br>2. Poultice, sap of the Leaves applied directly to affected area.	1. Diarrhea, Stomach pain, Inflammation<br>2. Haemostatic	Bejar184, CORD19
Pelargonium roseum Willd.	Malva Rosa	Whole plant, fresh	Oral	Horchata put 1Tbsp in 1L water.	Stomach pain	CORD7

Family/Species	Indigenous name	Plant part used	Admin.	Preparation	Use	Coll. #
Pelargonium zonale (L.) L'Herit.	Geranio	Whole plant, fresh	Oral	2 Tbsp in 1L of boiling water.	Cures wounds	CORD21
IRIDACEAE						
Sisyrinchium sp.	Pimienta del Campo	Whole plant, fresh	Oral	Horchata - 1 Tbsp in 1L boiling water with honey.	Desinflammatory, Stomach pain	CORD5
LAMIACEAE						
Melisa officinalis L.	Toronjil	Whole plant, fresh or dried	Oral	2 Tbsp in 1L of Horchata, boil slightly.	Nerves, Nervous system, Sadness, Depression, Heart pain	CORD60, Bejar286
Mentha x piperita L.	Hierba Buena	Whole plant, fresh	Oral	Cooked with sugar, Toronjil, Malva Olorosa.	Stomach ache	Bejar146
Mentha spicata L.	Hierba Buena	Whole plant, fresh	Oral	Horchata, For Horchata put 1 Tbsp of the whole plant in 1L of boiling water.	Stomach ache	CORD16
Mentha viridis L.	Menta	Whole plant, fresh	Oral, Topical	Boiled in waterfor worms, Boiled in milk for cold, Boiled with Aguardiente and rubbed on fro rheumatism.	Anthelmintic, Colds, Rheumatism	Bejar194
Minthostachys mollis Griesebach	Poleo	Leaves, fresh	Oral, Topical, Inhalation	Crush Leaves, inhale oils or boil.	Cold, Flu	Bejar250
Ocimum basilicum L.	Albaca	Whole plant, fresh	Oral	Cook, with Grama Dulce and Toronjil.	During childbirth	Bejar26
Rosmarinus officinalis L.	Romero	Whole plant, fresh	Oral, Topical	Cooked, with Aguardiente.	Bad Air / Mal Aire, Nerves, Sore eyes	Bejar254
Salvia microphylla Kunth.	Camotillo	Leaves and Flowers, fresh	Topical	With Aguardiente.	Inflammation of the arms and feet, Rheumatism	Bejar56
Salvia ramicifolia H.B.K	Salvia Real	Leaves and Flowers, fresh	Topical	With Aguardiente.	Rheumatism	Bejar272
Scutellaria sp.	Monte Negro	Whole plant, dried and ground	Oral and Topical	Grind together with Sangorache to get the extract. Put 4 tablespoons in 1/2l of lukewarm water. It should be drunk and rubbed in the body.	Headache, Fever	CORD17
Stachys sp.	Poleo Grande	Whole plant, fresh	Oral	Horchata, For Horchata put 1 Tbsp of the whole plant in 1L of boiling water.	Stomach pain, Stomach gases, Inflammations	CORD4
LAURACEAE						
Ocotea floribunda (Sw.) Mez.	Ishpingo	Whole plant, fresh	Topical	Rubbed with Monte de Coche, Chin Cil, Chila Chilo, Aguardiente.	Bad Air / Mal Aire, Skin irritation, Fright (Children) / Espanto caused by the rainbow	Bejar170
Ocotea sp.	Alcanfora	Whole plant, fresh	Topical	Rubbed with Monte de Coche, Chin Cil, Chila Chilo, Aguardiente.	Bad Air / Mal Aire, Skin irritation, Fright (Children) / Espanto caused by the rainbow	Bejar28
Persea americana Mill.	Palta	Seed, fresh	Oral	Crushed and boiled. 1. with sugar, 2. w/o sugar.	1. Abdominal pain 2. Toothache	Bejar228

Family/Species	Indigenous name	Plant part used	Admin.	Preparation	Use	Coll. #
LILIACEAE						
Allium cepa L.	Cebolla Blanca	Tuber, fresh	Oral	Expres juice and drink	Cough	Bejar70
LOGANIACEAE						
Buddleja americana L.	Salvia	Leaves, fresh	Topical	Cooked, with Aguardiente, as rubbing or bath.	Bad Air / Mal Aire, Headache, Eye sight	Bejar258
LORANTHACEAE						
Gaiadendron punctatum (Ruiz & Pav.) G. Don	Guajuro amarillo	Flowers, fresh	Oral	2 Tbsp of Flowers in 1L Horchata.	Yellow Fever	CORD72
LYCOPODIACEAE						
Huperzia brevifolia (Grev. & Hook) Holab	Agua Minga	Whole plant, fresh	Oral, Topical	3 Tbsp in 2L water for baths. 1 Tbsp for 1L Horchata.	Liver, Kidneys, Fever, Inflammation, Colds	CORD79
Huperzia columnaris B. Oellg.	Agua Minga	Whole plant, fresh	Oral, Topical	3 Tbsp in 2L water for baths. 1 Tbsp for 1L Horchata.	Liver, Kidneys, Fever, Inflammation, Colds	CORD84
Huperzia compacata (Hook.) Trevis.	Agua Minga	Whole plant, fresh	Oral, Topical	3 Tbsp in 2L water for baths. 1 Tbsp for 1L Horchata.	Liver, Kidneys, Fever, Inflammation, Colds	CORD83
Huperzia espinosana B. Oellg.	Agua Minga	Whole plant, fresh	Oral, Topical	3 Tbsp in 2L water for baths. 1 Tbsp for 1L Horchata.	Liver, Kidneys, Fever, Inflammation, Colds	CORD78
Huperzia hypogaea B. Oellg.	Agua Minga	Whole plant, fresh	Oral, Topical	3 Tbsp in 2L water for baths. 1 Tbsp for 1L Horchata.	Liver, Kidneys, Fever, Inflammation, Colds	CORD76
Huperzia kuestneri (Nessel) B. Ollg.	Agua Minga	Whole plant, fresh	Oral, Topical	3 Tbsp in 2L water for baths. 1 Tbsp for 1L Horchata.	Liver, Kidneys, Fever, Inflammation, Colds	CORD77, 81
Huperzia magellanicum (P. Beaum) Sw.	Agua Minga	Whole plant, fresh	Oral, Topical	3 Tbsp in 2L water for baths. 1 Tbsp for 1L Horchata.	Liver, Kidneys, Fever, Inflammation, Colds	CORD80
Lycopodium jusiaei Desv. ex Poir	Agua Minga	Whole plant, fresh	Oral, Topical	3 Tbsp in 2L water for baths. 1 Tbsp for 1L Horchata.	Liver, Kidneys, Fever, Inflammation, Colds	CORD82
Lycopodium vestitum Desv. ex Poir.	Agua Minga	Whole plant, fresh	Oral, Topical	3 Tbsp in 2L water for baths. 1 Tbsp for 1L Horchata.	Liver, Kidneys, Fever, Inflammation, Colds	CORD75
LYTHRACEAE						
Cuphea carthagenensis (Jacq.) J.F. Macbr	San Antonio	Whole plant, fresh	Topical	Externally as a bath or wash like sulfur to cleanse wounds.	Wounds	Bejar262
Cuphea loxensis Kunth.	Hierba del Toro	Whole plant, fresh	Oral	Cooked.	Leukorrhea	Bejar160
Cuphea racemosa (L.f.) Spreng.	Hierba del Coche	Whole plant, fresh	Topical	Cooked with Chila Chilo, Chin Chil, and Monte de la Huanchaca as a poultice.	Fright / Espanto (Children)	Bejar150
MALVACEAE						
Malva sylvestris L.	Malva Blanca	Whole plant, fresh	Oral	Cooked.	Laxative	Bejar182
Sida rhombifolia L.	Guintun verde	Leaves, fresh	Topical	Put the Leaves with salt and use for poultices, put on infections.	Infections	CORD31

Family/Species	Indigenous name	Plant part used	Admin.	Preparation	Use	Coll. #
MYRISTICACEAE						
Myristica fragrans Hout.	Nuez Moscada	Fruits, fresh	Topical	Apply with Aguardiente, Toronjil, Huanil del Campo, Hierba Mora, Payachilla, Mus Mus.	Bad Air / Mal Aire, Sorcery, Wounds (disinfection)	Bejar210
MYRTACEAE						
Eucalyptus globulus Labill.	Eucalipto	Leaves, fresh	Oral	Cooked with sugar Prenadilla and Orange Juice	Sore throat, Bronchitis	Bejar130
Psidium guineense Sw.	Guayavilla	Yellow Fruits and Flowers, fresh	Oral	Drink juice and Flowers extract with sugar.	Diarrhea	Bejar142
NYCTAGINACEAE						
Mirabilis jalapa L.	Buenas Tardes	Root, fresh	Oral	Cooked with San Agustin Yullo.	Purgative	Bejar46
ONAGRACEAE						
Fuchsia loxensis H.B.K.	Pena Pena	Whole plant, fresh	Oral	1/2 Tbsp with 1L of Horchata.	Nerves	CORD10
Oenothera rosea Aiton	Shullo	Whole plant, fresh	Oral	horchata - take 1 Tbsp of the whole plant an put in 1L water, add honey.	Liver, Kidneys	CORD26
ORCHIDACEAE						
Epidendrum acrorhodum Hagster & Dodson	Hierba del Caballero	Flowers, fresh	Oral	With cold water, sugar, Chirimoya, Orange and Lime Flowers, Toronjil, Congona, white roses and some drops of Aguardiente.	Fragrance, Good Luck	RBU/PL340
Epidendrum cochlidium Lindl.	Flor de Christo	Flowers, fresh	Oral	With cold water, sugar, Chirimoya, Orange and Lime Flowers, Toronjil, Congona, white roses and some drops of Aguardiente.	Nerves	Bejar132
Lycaste gigantea Lindl	Caña Caña	Flowers, fresh	Oral	Boiled with Cola de Caballo, Llanten, Matico Culantrillo and Cadillo.	Kidneys	Bejar60
	Simayuca	Fruits, fresh	Oral	Boiled with Preñadilla and Guayusa.	Male fertility	Bejar276
OXALIDACEAE						
Oxalis peduncularis H.B.K.	Trebol	Whole plant, fresh	Oral	Cooked with Sauco, lemon and bismocarbonato.	Diarrhea, Scurvy	Bejar288
PAPAVERACEAE						
Argemone mexicana L.	Cardo Santo, Anís	Whole plant, fresh	Oral	Boil to extract the juice, take with sugar, 1 Tbsp in 1L tea.	Sore eyes, Lungs, Fright / Susto, Stomach pain, Stomach inflammation	Bejar64, CORD67
Papaver somniferum L.	Amapola Roja, Amapola Blanca	Flowers, fresh	Oral	1 Tbsp of Flowers in 1L of Horchata.	Purify the blood, Vaginal discharge with blood, Menstrual regulation	CORD56. Bejar30, 32
PASSIFLORACEAE						
Pasiflora ligularis Jus.	Grenadilla	Peel of Fruits, Leaves, fresh	Topical	Cooked, applied as poultice with agua florida.	Infections, Menstrual cramps	Bejar140

Family/Species	Indigenous name	Plant part used	Admin.	Preparation	Use	Coll. #
PIPERACEAE						
Peperomia alata R.&P.	Congona Serrana	Whole plant, fresh	Oral	Refrigerated, Take 1 spoon of refrigerated mixture in lukewarm water. for nerves taken together with "Pega Pega" (Fuchsia loxensis). for Evil Wind and much sweating spray on body. It doesn't work as well as Congona. .	Evil wind / Mal viento, Heart, Nerves	CORD13
Peperomia blanda (Jaq.) H.B.K.	Congona	Whole plant, fresh	Oral	Refrigerated, Take 1 spoon of refrigerated mixture in lukewarm water. for nerves taken together with "Pega Pega" (Fuchsia loxensis). for Evil Wind and much sweating spray on body.	Evil wind / Mal viento, Heart, Nerves. It doesn't work as well as Congona	CORD12
Peperomia congona Ruiz & Pav.	Congona	Whole plant, fresh	Oral	Cooked.	Nerves, Heart	Bejar86
Piper aduncum L.	Cordoncillo, Matico	Whole plant, fresh	Oral, Topical	For the liver 3 Tbsp in 1L Horchata, drink with sugar, Grama Dulce, Caña agria and Cola de Caballo. for washing wounds.	Liver, Wounds	Bejar88, 192. CORD58
Piper angustifolium L.	Cordoncillo, Matico	Whole plant, fresh	Oral, Topical	For the liver 3 Tbsp in 1L Horchata, drink with sugar, Grama Dulce, Caña agria and Cola de Caballo. for washing wounds.	Liver, Wounds	Bejar88, 192. CORD58
PLANTAGINACEAE						
Plantago australis Lam.	Llantén	Whole plant, fresh	Oral	Cooked, taken with Huichinge, Cola de Caballo and Mortiño.	Liver, Kidneys	Bejar178
Plantago major L.	Llantén	Whole plant, fresh	Oral	Horchata, 1 Tbsp in 1L Horchata.	Liver, Breast pain, Anti Inflammatory	CORD64
POACEAE						
Bromus pitensis Kunth.	Triguillo	Whole plant, fresh	Topical	Cooked and rubbed externally.	Urinary infections, Woman problems	Bejar292
Cymbopogon citratus (DC.) Stapf.	Hierba Luisa, Paja Luisa	Leaves and Root, fresh	Oral	Use root for dysenteria and Leaves to make tea.	Dysenteria	CORD20
Cynodon dactylon (L.) Pers.	Grama Dulce	Whole plant, fresh	Oral	Cooked, taken with Toronjil, Caña Agria, Cadillo and sugar.	Kidneys	Bejar138
Holcus lanatus L.	Abrecaminos, Hierba del Perro	Whole plant, fresh	Oral	Cooked, with Malva Olorosa, Paico, Agua de Huayaddilla, and sugar.	Diarrhea occurring with a cold	Bejar154
Hordeum vulgare L.	Cebada	Seeds	Oral	1. Seeds are cooked and pased through a strainer, taken in the afternoon.<br>2. Seeds (grains), no fever present toasted grains with Oregano and white onions in water.	1. Tiredness<br>2. Diarrhea	Bejar68

Family/Species	Indigenous name	Plant part used	Admin.	Preparation	Use	Coll. #
Melinis minutiflora P.Beauv.	Yaragua	Whole plant, fresh	Oralen	Feed.	Good fodder for cows and guinea pigs, etc.	CORD27
POLEMONIACEAE						
Cantua quercifolia Jus.	Pepiso	Leaves, fresh	Topical	Parboiled, as bath or rubbing.	Rheumatism	Bejar238
POLYGONACEAE						
Polygonum hydropiperoides Michaux	Soliman, Solimancillo	Whole plant, fresh	Topical	Put some plants in 2L boiling water and sometimes add Matico and Geranio.	Pimple, Wash wounds of livestock	CORD63
POLYPODIACEAE						
Polypodium spp.	Calahuala	Root, fresh	Oral	Cooked, taken with Honey, Grama Dulce and .	Kidneys	Bejar52
PORTULACACEAE						
Portulaca oleracea L. subsp. *tuberculata* Danin & H.G. Baker	Cutuyuyo, Verdalonga	Whole plant, fresh	Topical, Oral	Crushed and boiled, eaten fresh with salsa and oil or rice.	Fever, Liver	Bejar94, 300
PROTEACEAE						
Oreocallis grandiflora (Lam.) R.Br.	Boldo	Flowers, Leaves, fresh	Oral	Horchata, take 1 Tbsp of Flowers and Leaves an put in 1L of boiling water.	Liver, Dizziness, Cirrhosis, Wash the blood	CORD3
Roupala loxensis I.M. Johnst.	Tarro Serrano	Whole plant, fresh			Dye yarn brown, Flavor Orals	CORD85
PUNICACEAE						
Punica granatum L.	Granada	Leaves, fresh	Oral	2 Tbsp in 1L of boiling water.	Dysenteria, Diarrhea with blood	CORD55
ROSACEAE						
Eriobotrya japonica (Thunb.) Lindl.	Nispero	Leaves, fresh	Oral	Boiled.	Liver, Reduce Cholesterol, Diabetes	Bejar208
Sanguisorba officinalis L.	Pimpinella de Bejuco	Whole plant, fresh	Topical	Rubbed exterenally.	Bad Air / Mal Aire	Bejar244
RUBIACEAE						
Cinchona officinalis L.	Cascarilla	Bark, dried	Oral	Boil 1/2 Tsp in 1L water. in Aguardiente 1 Tbsp for 1/2l .	Freight/ Susto, Fever, Bone pain, Malaria, Malaria Tertiana, Weight loss, Flu or colds	CORD6, Bejar66
RUTACEAE						
Citrus aurantium L.	Naranja Agria	Fruits, Peel, fresh	Oral	Juice of two Fruits with Guayusa as vitamin supplement for breakfast, Peel burned as incense in ceremonies.	Scurvy, Stomach ache, High blood pressure, Deodorant, Bad Air / Mal Aire	Bejar206
Ruta graveolens L.	Ruda	Whole plant, fresh	Topical	Used as poultice under arms, heated naturally. for baths and frotations: 2 Tbsp in 1/2L of Aguardiente, 1/2 Tbsp in 1/2L of water.	Sore eyes, Bad humor, Dizziness, Headache, Nausea, Vomiting, Cleansing/ Limpiar	Bejar256, CORD25

Family/Species	Indigenous name	Plant part used	Admin.	Preparation	Use	Coll. #
SALICACEAE						
Salix humboldtiana Willd.	Sauce	Leaves, fresh	Oral	1. Boiled with Aguardiente<br>2. Chewed	1. Fever<br>2. Infections	Bejar268
SAPINDACEAE						
Dodonaea viscosa Jacq.	Chamana	Whole plant, fresh	Topical	Macerated in Aguardiente, 3 times per week, every other day.	Arthritis, Bones, Cold, Muscle pain, Bone pain, Rheumatism	Bejar72, 96
SCROPHULARIACEAE						
Scoparia dulcis L.	Tiatina	Whole plant, fresh	Topical	Wash affected area and apply as a poultice.	Bruises caused by physical blows to the body	Bejar284
SELAGINELLACEAE						
Selaginella arthritica Alston	Doradilla, Trensita, Trensilla	Whole plant, fresh	Oral	Cooked, taken with sugar, Chuquiragua, Verbena, Canchalagua.	Bones, sore bones	Bejar126
SOLANACEAE						
Acnistus arborescens (L.) Schltdl.	Pico Pico	Leaves, fresh	Oral	Boil, give a large glass with Mosquera, Chirimoya and a lot of sugar. if patient doesn't vomit, give him another glass.	Stomach, Vomiting, Purgative, Stomach swelling	Bejar242
Brugmansia candida Persoon	Floripondio, Guando	Leaves, Flowers, fresh	Oral		Freight / Susto	CORD90
Cestrum auriculatum L'Herit	Sauco Comun	Whole plant, fresh	Oral, Topical	Cook and mixed with Trebol, lemon and bismocarboanate, drink or rub externally.	Diarrhea, Scurvy	Bejar270
Cestrum sp.	Sauco Verde	Leaves, fresh	Topical	Put the juice of the leaves in water, put on forehead	Against heat/fever	CORD68
Datura ferox L.	Chamico	Leaves, fresh	Topical	Remove leaves and then rub affected area with them.	Asthma, Rheumatism	Bejar98
Iochroma loxense (Kunth.) Miers.	Marapico	Whole plant, fresh	1. Topical, 2.,3.Oral	1. Applied as ointment,<br>2. Flowers boiled with Flor de Christo, Clavel, Pega Paga<br>3. Crushed and boiled with Pega de Algodon, steer haunch and Zapallo Serrano.	1. Toothache.<br>2. Nerves<br>3. Tumors	Bejar188
Nicotiana tabacum L.	Tabaco	Whole plant, fresh	Topical	Rub externally.	Parasites, Fungicide, Insecticide	Bejar282
Solanum americanum Mill.	Mortiño, Hierba Mora, Yerba Mora	Whole plant, fresh	1. Oral, 2. Topical	1. 1 Tbsp in 1L boiling water<br>2. Crush Leaves and apply on skin.	1. Fatigue of drunkards, Nausea<br>2. Sorcery	CORD49, Bejar158
Solanum albidum Dunal	Tululuche	Whole plant, fresh	Topical	Rub externally.	Rheumatism	Bejar294
Solanum nigrum L.	Mortiño	Whole plant, fresh	Oral	Cooked, taken with aspirin and sugar.	Kidneys, Hangover fever, Vomiting, Shame	Bejar202

Family/Species	Indigenous name	Plant part used	Admin.	Preparation	Use	Coll. #
Streptosolen jamesonii (Benth.) Miers	Arco Iris, Flor de Quinde	Whole plant, fresh	1. Topical<br>2. Oral	Poleo de Gentil, 7 Espiritús, and Timolina.	1. Fright (Children)/ Espanto, Yellow fever<br>2. Heart attack, Epilepsia	Bejar38, CORD66
TILIACEAE						
Triumfetta mollissima Lam.	Cadillo	Whole plant, fresh or dried	Oral	Crushed and cooked for Horchata, 3 Tbsp in 1L Horchata. with sugar, phospherous tablets (Aspirin), Caña Agria, Cola de Caballo, Trensilla and Culantrillo.	Kidneys, Inflammation	CORD48, Bejar50
URTICACEAE						
Parietaria debilis G. Forst.	Palitalia	Whole plant, fresh	Oral	Crushed and boiled with Aguardiente, salt and Naranja Agria, taken for nine days.	Internal bleeding caused by a blow	Bejar224
Pilea microphylla (L.) Lieberman	Preñadilla	Whole plant, fresh	Oral	Cook, mix with Simayuca, Guayusa, Aromatic Eucalyptus, and Sugar, take once daily for one month.	Fertility, Diuretic, Lungs, Venereal diseases	Bejar252
***Urtica dioica* L.**	Ortiga Negra	Whole plant, fresh	Topical	Rubbed externally: affected area should be rubbed with alcohol or iodine afterwards.	Muscle pain	Bejar218
Urtica magellanica Juss. ex Poir.	Ortiga Blanca	Whole plant, fresh	Topical	Rubbed externally: affected area should be rubbed with alcohol or iodine afterwards.	Muscle pain	Bejar212
***Urtica urens* L.**	Chige, Ortiga	Whole plant, fresh	Oral	Cooked.	Neurological pain, Purify the blood	CORD11, Bejar50
Urera baccifera (L.) Gaudich ex Wedd.	Ortiga de Caballo	Whole plant, fresh	Topical	Rubbed externally: affected area should be rubbed with alcohol or iodine afterwards.	Muscle pain	Bejar214
Urera spp.	Ortiga de Leones del Oriente	Whole plant, fresh	Topical	Rubbed externally: affected area should be rubbed with alcohol or iodine afterwards.	Muscle pain	Bejar216
VALERIANACEAE						
Valeriana convallarioides (Schmale) B.B. Larsen	Valeriana Grande, Lirio	Whole plant, fresh	Oral	1 Tbsp in 1L boiling water.	Yellow Fever	CORD54
Valeriana decussata Ruiz & Pav.	Valeriana	Bark and Root, fresh	Oral	Soaked in Aguardiente with sugar, Toronjil, and Congona.	Heart, Nervous system, Nerves	Bejar298
Valeriana microphylla H.B.K.	Valeriana Grande Rosada	Whole plant, fresh	Oral	2 Tbssp in 1L boiling water or Aguardiente.	Bone pain, Cramps, Evil Air / Mal Aire, Kidney	CORD73
Valeriana plantaginea H.B.K.	Valeriana, Lirio pequeño	Whole plant, fresh	Oral	2 Tbssp in 1L boiling water or Aguardiente.	Bone pain, Cramps, Evil Air / Mal Aire, Kidney	CORD74
Valeriana sp.	Violeta Serrana	Whole plant, fresh	Oral	1 Tbsp in 1L Horchata.	Cough	CORD40
VERBENACEAE						
Lantana communis L.	San Agustin Lluyo	Root, fresh	Oral	Boiled, take with sugar and Buenas Tardes.	Purgative	Bejar260

Family/Species	Indigenous name	Plant part used	Admin.	Preparation	Use	Coll. #
ZINGIBERACEAE						
Costus glabratus S.W.	Caña Agria	Whole plant, fresh	Oral	Boiled with Cola de Caballo, Llanten, Matico Culantrillo and Cadillo.	Kidneys, Diabetes	Bejar58
INDET.						
	Hierba de Venado	Whole plant, fresh	Oral	Timolina.	White and yellow vaginal discharge, Inflammation	CORD35
